# Sonothrombolysis: State-of-the-Art and Potential Applications in Children

**DOI:** 10.3390/children11010057

**Published:** 2023-12-31

**Authors:** Rebecca E. Ward, Santiago Martinez-Correa, Luis Octavio Tierradentro-García, Misun Hwang, Chandra M. Sehgal

**Affiliations:** 1 Department of Radiology, Children’s Hospital of Philadelphia, Philadelphia, PA 19104, USA; rebecca.ward@pennmedicine.upenn.edu (R.E.W.); martinezcs@chop.edu (S.M.-C.); l.tierrandentrogarci@mwmc.com (L.O.T.-G.); hwangm@chop.edu (M.H.); 2 Department of Radiology, Perelman School of Medicine, University of Pennsylvania, Philadelphia, PA 19104, USA

**Keywords:** ultrasound, sonothrombolysis, diagnostic, therapeutic, pediatric

## Abstract

In recent years, advances in ultrasound therapeutics have been implemented into treatment algorithms for the adult population; however, the use of therapeutic ultrasound in the pediatric population still needs to be further elucidated. In order to better characterize the utilization and practicality of sonothrombolysis in the juvenile population, the authors conducted a literature review of current pediatric research in therapeutic ultrasound. The PubMed database was used to search for all clinical and preclinical studies detailing the use and applications of sonothrombolysis, with a focus on the pediatric population. As illustrated by various review articles, case studies, and original research, sonothrombolysis demonstrates efficacy and safety in clot dissolution in vitro and in animal studies, particularly when combined with microbubbles, with potential applications in conditions such as deep venous thrombosis, peripheral vascular disease, ischemic stroke, myocardial infarction, and pulmonary embolism. Although there is limited literature on the use of therapeutic ultrasound in children, mainly due to the lower prevalence of thrombotic events, sonothrombolysis shows potential as a noninvasive thrombolytic treatment. However, more pediatric sonothrombolysis research needs to be conducted to quantify the safety and ethical considerations specific to this vulnerable population.

## 1. Introduction

Ultrasound is fundamental to the field of pediatrics. This portable and cost-effective diagnostic tool is crucial in diagnosing emergent pediatric ailments, such as pyloric stenosis, appendicitis, testicular torsion, and germinal matrix hemorrhages. Congenital anomalies, such as hip dysplasia, posterior urethral valves, and spinal dysraphism, are also routinely diagnosed via ultrasound. In addition to its low cost and portability, ultrasound is the preferred method of diagnostic imaging for children and neonates due to the lack of harmful radiation compared with other modalities, such as CT, fluoroscopy, and conventional X-ray. Given its preferable imaging characteristics, pediatric interventionalists rely heavily upon ultrasound for various procedures, such as vascular access, lumbar puncture, abscess drainage, and paracentesis. In recent years, advances in ultrasound therapeutics have been implemented in treatment algorithms for the adult population. In children, sonothrombolysis has been utilized to treat pulmonary artery obstruction after failed catheter-directed thrombolysis with alteplase, showing successful results [1].

Arterial and venous thrombotic events are associated with a high rate of morbidity and mortality [2]. Although thrombectomy, tissue plasminogen activator (tPA), and anticoagulation remain the mainstay of thrombolysis, ultrasound-guided thrombolysis has emerged as a promising technology. Often used in conjunction with other therapies, such as tPA, sonothrombolysis utilizes ultrasound waves to mechanically disrupt clots. The addition of microbubble contrast agents to sonothrombolysis has further improved recanalization rates [3]. Despite gaining more popularity in the past decade, the origin of sonothrombolysis dates back to the 1960s, with a publication by Anschuetz and Bernard suggesting sonothrombolysis as a treatment for atherosclerotic disease [4]. In 1976, Trübestein et al. created artificial thrombi in the iliac and femoral arteries and veins of forty-four dogs. Their study determined that sonothrombolysis was capable of destroying thrombus within two to five minutes while maintaining the architectural integrity of the vessel wall [5]. Approximately 20 years later, in 1993, Siegel et al. published a small patient cohort study where percutaneous ultrasound angioplasty was determined to be useful for recanalization of fibrous thrombotic arterial occlusions, with a restenosis rate of 20 percent at 6–12 months [6,7]. Around the same period, Sehgal and colleagues demonstrated the combined use of intravascular ultrasound with thrombolytic agents to increase clot lysis [8,9].

More recently, sonothrombolysis research has primarily focused on treatment for pulmonary embolism, ischemic stroke, myocardial infarction, deep venous thrombosis, and peripheral arterial occlusion in conjunction with microbubble contrast and other lysis agents, such as tPA [10,11,12,13]. Overall, these studies and case reports have demonstrated further increased clot disaggregation with the addition of ultrasound than with a single thrombolytic agent alone, ultimately leading to improvement in treatment efficiency and safety parameters. However, despite its promising applications, the use of therapeutic ultrasound in the pediatric population remains poorly defined. This review aims to quantify the use of therapeutic ultrasound, specifically sonothrombolysis, in pediatric research and clinical care.

## 2. Materials and Methods

For this review paper, we conducted a Pubmed literature search to obtain clinical and preclinical studies that detailed the use and applications of sonothrombolysis, only including articles published before February 2023. The following search terms were employed: “sonothrombolysis” (Title/Abstract) OR “ultrasound thrombolysis” (Title/Abstract) OR “sonographic thrombolysis” (Title/Abstract) OR “ultrasound-assisted thrombolysis” (Title/Abstract).

**Figure 1:**
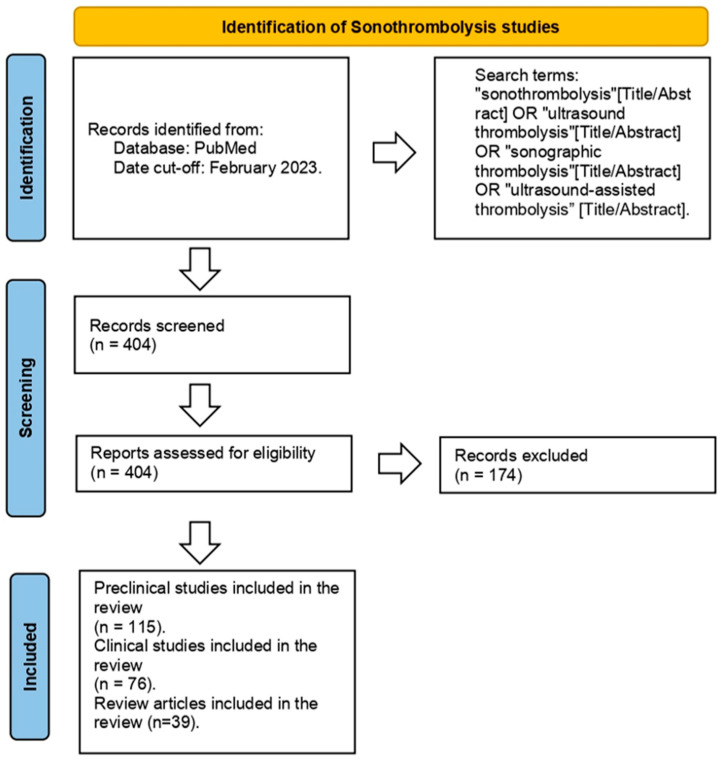
Identification process of sonothrombolysis studies.

**Figure 2:**
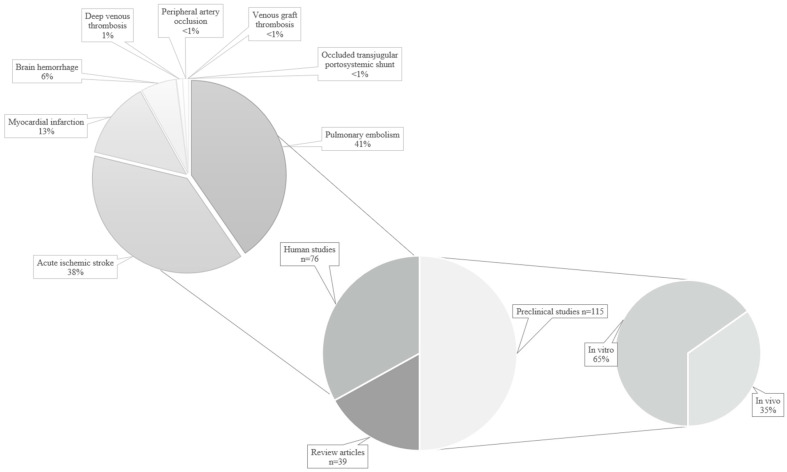
Type of studies included in the revision.

Three reviewers (RW, LOTG, and SMC) individually screened 404 papers by title and abstract to include original articles describing sonothrombolysis in preclinical or clinical settings. We summarized the article identification process using a PRISMA-like flow diagram (Figure 1) [14]. Key articles are highlighted in Table 1. Other relevant sonothrombolysis articles that were not identified in the original Pubmed search were screened and included if the search criteria were fulfilled. Studies published after February 2023, or with a focus other than sonothrombolysis, were excluded. After initial screening, we obtained 230 original articles, 39 of which were classified as review articles. Of the remaining articles, 115 were preclinical trials, including in vitro (*n* = 75) and in vivo (*n* = 40) studies. A total of 76 studies were conducted in humans (Figure 2). For human studies, we extracted the age group, the condition to be treated, the country where the study was performed, and the number of patients who were treated. These details are summarized in Table 2.

## 3. Physics Principles

Sonothrombolysis can be performed with ultrasound alone, with ultrasound and a thrombolytic agent, or with ultrasound and microbubbles, working through the following mechanisms: heat, mechanical effects, augmentation with acoustic cavitation, and microbubbles.

### 3.1. Thermal Effects

The thermal effects of sonothrombolysis on clot disaggregation are not definitive. In vivo, sound energy attenuated by a patient’s tissues is converted to other forms of energy, predominantly heat, resulting from the law of the conservation of energy. Because of this generated thermal energy, providers avoid scanning delicate tissue, such as fetuses, for prolonged periods to avoid adverse events. With respect to sonothrombolysis, increasing the temperature also intensifies tPA enzymatic activity, which in turn expedites thrombolysis [28]. This phenomenon is important when tPA is used in conjunction with sonothrombolysis, as tPA shows temperature dependence in vitro [29]. Conversely, some studies suggest that thermal variables play less of a role in sonothrombolysis. In several in vitro studies, temperature during sonification was maintained at 37 °C, suggesting that sonothrombolysis occurred via other mechanisms [30]. Shaw et al. concluded that sonothrombolysis lytic efficacy was not significantly reduced at 33 °C in an in vitro human clot model, also supporting nonthermal-dependent thrombolysis [31].

### 3.2. Mechanical Effects

Ultrasound acoustic radiation force is the transfer of momentum in acoustic waves, which can be used to disrupt tissue and manipulate cells [32]. During sonothrombolysis, acoustic radiation force (ARF) causes local displacement across the clot surfac in addition to widening the clot pores to facilitate transport of thrombolytic drugs into the thrombus. The increased efficacy of the thrombolytic agent via ARF lowers the required dosage. A lower thrombolytic dose in turn reduces the risk of hemorrhage and other complications. Acoustic radiation force also creates motion in the blood surrounding the thrombus, a process called acoustic streaming, which increases clot permeation and facilitates thrombus penetration by the thrombolytic drug [33]. Additional fibrin-binding sites are also exposed to plasmin at the area of thrombus via acoustic streaming [34].

### 3.3. Acoustic Cavitation

Acoustic cavitation refers to the formation of oscillating bubbles by acoustic pressure. Cavitation is defined as inertial or stable. During inertial cavitation, the bubble collapses, producing a current on the clot surface, which damages the clot and exposes binding sites for thrombolytic agents. Stable cavitation refers to milder bubble oscillation, which also generates mechanical damage as the bubbles within the thrombus expand and contract during ultrasound. With the addition of a microbubble contrast agent, the likelihood of cavitation increases [33,35]. The mechanical index, which will be discussed in greater detail later, is a marker for an ultrasound beam’s ability to cause cavitation-related effects, which essentially reflects tissue mechanical damage [36].

### 3.4. Microbubbles

Microbubbles are tiny gas- or air-filled microspheres that serve as ultrasound contrast agents, ranging in size from 2 to 10 µm and containing a lipid, polymer, or lipopolymer shell [37]. In addition to high molecular weight/low solubility gases, microbubble shells can also be loaded with fluorophores, genes, or drugs [38]. During diagnostic ultrasound examinations, microbubbles generate an acoustic impedance mismatch between body tissue and fluid, which enhances the reflection of sound [39]. Clinically, FDA-approved contrast agents are most suitable for low-frequency examinations, typically between 5 and 10 MHz. These agents include Sonovue/Lumason, Definity/Luminity, and Sonazoid [40]. In the pediatric population, ultrasound contrast agents are widely used, such as for the evaluation of vesicoureteral reflux, focal liver lesions, and blunt abdominal trauma, in addition to mass lesions and inflammation in abdominal and pelvic organs. Recently, contrast agents have been used for the assessment of Crohn’s disease and cerebral ischemia [41].

Multiple studies have demonstrated that microbubble contrast agents and ultrasound exposure increase the degree of drug-mediated thrombolysis [42,43]. A study by Cintas et al. demonstrated that galactose-based microbubbles (Levovist) strongly accelerated lysis of thrombus when used with low-frequency high-power ultrasound and recombinant tPA. By serving as cavitation nuclei, microbubbles lower the energy required for acoustic cavitation [44]. A study by Molina et al. determined that microbubble-enhanced ultrasound increased thrombolysis in acute ischemic stroke, resulting in improved arterial recanalization and better patient short- and long-term outcomes [39]. Similarly, high-intensity ultrasound (10 W/cm^2^) with microbubbles produced significantly higher patency and flow scores than ultrasound with saline in patients with arteriovenous graft thrombi [45]. Mathias and colleagues utilized transthoracic high-mechanical index impulses (1.7 MHz, mechanical index 1.3) combined with a microbubble contrast agent as a compassionate measure to insonify the upper and superior part of the inferior left pulmonary lobes in a 13-month-old girl with a thrombotic occlusion of the left pulmonary artery. After 50 min, they confirmed recanalization and the patient had an improvement in systemic oxygenation [1].

## 4. Acoustic Parameters

Several ultrasound systems are commercially available for sonothrombolysis. However, the acoustic parameters used to achieve sonothrombolysis often vary between machines. In 2014, Zhou and Ramaswami compared three commercially available ultrasound diagnostic systems, Siemens Sequoia, Philips iU22, and Sonosite Micromaxx, using an in vitro bovine blood clot model. Each system was programmed with a different central frequency, focal length, spatial peak pulse average acoustic intensity, microbubble destruction, mechanical index, rarefactional pressure, and pulsed wave parameter. Despite differences in acoustic field characteristics, all three devices demonstrated comparable satisfactory outcomes when used in conjunction with tPA and microbubbles for in vitro thrombolysis [46].

Multiple review articles have compiled acoustic parameter results from preclinical and clinical sonothrombolysis studies. A study by Bor-Seng-Shu et al. reviewed randomized clinical studies that coupled sonothrombolysis with tPA for treatment of acute ischemic stroke in adult humans. Four trials of small sample size were ultimately included in the study and delineated into the following groups: tPA only, tPA with ultrasound, and tPA with ultrasound and microbubbles. The ultrasound frequencies ranged from 1.8 to 2 MHz, and the duration of sonographic ultrasound exposure spanned from 60 to 120 min. Despite the heterogeneity in acoustic parameters and sonothrombolysis protocols, Bor-Seng-Shu et al. concluded that ultrasound thrombolysis with tPA is a safe procedure that increases the rate of recanalization in ischemic stroke. Additionally, at these sonographic parameters, the combination of tPA and sonothrombolysis did not increase the risk of symptomatic intracranial hemorrhage [47].

Papadopoulos et al. also reviewed sonothrombolysis protocols and compiled various study parameters for preclinical in vitro, preclinical animal, and clinical human studies [30]. In the in vitro studies reviewed, most of which used a human model, intensities ranged from 0.5 W/cm^2^ to 193 W/cm^2^, while frequencies varied from 20 KHz to 2 MHz. The most common frequency recorded was 1 MHz. Further, the most frequently used thrombolytic agent was recombinant tPA, with a concentration ranging from 0.1 to 100 µg/mL and a treatment time ranging from 0.5 to 720 min. In preclinical animal models reviewed by Papadopoulos et al., most commonly employing rabbits, frequencies ranged from 20 kHz to 5.7 MHz, with the most used frequency again recorded as 1 MHz. The most typical thrombolytic agent was tPA, with drug concentrations ranging from 0.8 to 10 µg/mL, significantly lower than the dosages used in the in vitro experiments. Treatment times ranged from 2 to 120 min, with most studies reporting 60 min of treatment time. Some of the included animal studies demonstrated increased sonothrombolysis efficiency with the inclusion of microbubbles. Finally, in adult human trials reviewed by Papadopoulos et al., all of which evaluated thrombus within the middle cerebral artery (MCA), frequencies ranged from 300 kHz to 4 MHz. All included trials used recombinant t-PA, with some of the selected studies including microbubble administration. The dosage of tPA was a standard 0.9 µg/mL, with treatment times ranging from 10 to 30 min. Efficacy was also reviewed, and, similar to other studies, it was concluded that ultrasound increases the ability of a thrombolytic agent to bind to a clot and assists in thrombus disaggregation [30].

Another review study using an in vitro human blood clot model determined that optimal clot lysis effects occurred between acoustic pressures of 500 and 600 kPa when used in conjunction with microbubbles [48], another important parameter to consider during in vitro and in vivo trials.

The acoustic behavior of microbubbles at different time points during prolonged tone burst excitation has also been explored. Chen et al. evaluated the acoustic behavior via direct high-speed optical observation and passive cavitation detection in a cellulose hollow fiber [17]. The group observed that remnant bubbles persisted as clusters oscillating with large amplitude during a single long tone burst. This observation may explain the superior therapeutic effect of long acoustic cycles in sonothrombolysis compared with shorter pulses. While there is no consensus on the optimal ultrasound parameters for sonothrombolysis, the observed success of sonothrombolysis with a broad spectrum of sonication conditions suggests the potential to treat pediatric patients.

## 5. Intravascular Sonothrombolysis

In addition to external standard ultrasound probes, invasive intravascular sonothrombolysis catheters demonstrate successful sonothrombolysis. Unlike a standard probe, an intravascular transducer can deliver ultrasound waves directly adjacent to thrombus. One in vivo and ex vivo study found that a forward-viewing intravascular transducer demonstrated no evidence of histological vessel damage or downstream clot particles >500 µm, suggesting that this transducer poses minimal risk [49]. Wu et al. developed a dual-mode ultrasound intravascular catheter device for imaging blood vessels and sonothrombolysis. The group created a dual-mode catheter by combining a 16 MHz high-frequency element (imaging transducer) with a low-frequency 220 kHz element (treatment transducer), assembling the transducers into a 10 Fr catheter. An in vitro bovine clot was significantly reduced in 30 min. By including the 16 MHz imaging transducer, the investigators could better position the treatment transducer for optimizing sonothrombolysis [50]. Although intravascular sonothrombolysis devices are still largely in developmental stages, the future application of these devices could transform the future of sonothrombolysis. Further investigation is needed to determine if intravascular sonothrombolysis is a safe and feasible option for neonates and children.

## 6. Preclinical In Vitro Studies

The efficacy of sonothrombolysis in occluded vessels has been widely examined during the last 20 years. These studies have mainly evaluated the mechanical properties of blood clots after sonothrombolysis therapy in controlled environments that intend to simulate biological systems and pathological conditions. In our search, most of the original studies in sonothrombolysis were preclinical. A total of 48 studies evaluated the efficacy of sonothrombolysis in different conditions, from which 28 were conducted in in vitro models. The majority of the in vitro studies evaluated the added value of sonothrombolysis to fibrinolytic therapy or the effect of ultrasound contrast agents on thrombolysis.

In 2013, Hölscher et al. tested the effect of different pulse width (PW) and duty cycle (DC) combinations on high-intensity focused ultrasound (HIFU)-induced sonothrombolysis efficacy by exposing artificial blood clots to pulsatile flow. An increase in thrombolysis efficacy could be seen in association with DC and/or PW for all study groups [51]. Ahadi and colleagues investigated the effect of increasing acoustic output powers on potential clot fragmentation using transcranial high-intensity focused ultrasound, demonstrating that sonothrombolysis can be executed effectively without causing clot fragmentation using acoustic output powers of less than 400 W [52].

Some authors have also studied the added value of laser to HIFU in improving thrombolysis efficacy. Cui et al. evaluated the feasibility of adding laser enhancement to HIFU in vitro treatment in bovine blood clots, showing that without the use of thrombolytic drugs or microbubbles, laser-enhanced HIFU thrombolysis could improve clot cavitation by dramatically lowering its threshold [53]. Similarly, Jo et al. developed a fiber optics-based laser-ultrasound thrombolysis device. They assessed the efficacy of this method for restoring blood flow in an in vitro flow clot model. Jo et al. combined an endovascular laser thrombolysis system with HIFU to remove the clot. Their model revealed that ultrasound threshold pressures for efficient thrombolysis decreased, suggesting that treatment efficiency and safety could be enhanced [15].

The use of ultrasound cavitation of microbubble contrast agents has been shown to be a potential tool in vascular occlusion therapy in different in vitro systems. A study conducted in 2015 assessed the ability to combine ultrasound and microbubbles with rtPA therapy to degrade fibrin in human blood clots [16]. Clot lysis was determined by diameter loss and release of radioactive fibrin. The researchers found that, at higher acoustic pressures where inertial cavitation is achieved, the combination of rtPA, ultrasound, and microbubbles enhanced fibrin degradation compared with rtPA therapy alone. However, no enhancement was observed at lower acoustic pressures (stable cavitation). In 2017, Goyal et al. evaluated the optimal ultrasound parameters for achieving microvascular reperfusion caused by microthrombi when combined with tPA [18]. Investigators utilized venous and arterial microthrombi in in vitro models to assess the specific effects of ultrasound, with or without tPA, in achieving microvascular reperfusion. A synergistic effect was found between internal cavitation regimes and tPA therapy, suggesting that this combination may be utilized to reach a higher degree of fibrinolysis of both thrombus types.

Recently, Auboire et al. assessed blood clots’ acoustic and elastic properties’ changes by measuring sound speed and shear wave speed under sonothrombolysis with microbubbles [19]. Human blood clots were submitted to a combination of microbubbles and rtPA in an in vitro setup. Investigators found that blood clots exposed to both tPA and sonothrombolysis became stiffer than those treated with tPA alone, which might be explained by platelet activation. Further research is sought to evaluate this finding using other approaches focused on platelet recordings.

## 7. Preclinical Animal Studies

Animal models have been crucial in understanding the role of sonothrombolysis in different conditions, particularly ischemic stroke, thrombosis, and cardiovascular conditions, which can be applicable to both adult and pediatric populations. Researchers have utilized different animal species, including mice, rats, rabbits, pigs, and nonhuman primates, to investigate the use of this technique in the aforementioned pathological conditions. We found 46 animal studies, from which 14 evaluated intracranial thrombosis [3,20,21,22,23,54,55,56,57,58,59,60,61,62], 24 extracranial thrombosis [57,63,64,65,66,67,68,69,70,71,72,73,74,75,76,77,78,79,80,81,82,83], and 8 other vascular and cardiovascular conditions [84,85,86,87,88,89,90,91].

### 7.1. Intracranial Thrombosis

Shimizu et al. examined the safety of transcranial-targeting midfrequency (0.1 to 1 MHz) ultrasonic thrombolysis for acute ischemic stroke in healthy Macaca monkeys in 2012 [23]. Investigators assigned nine young adult cynomolgus monkeys (5.9 ± 0.9 years old) to a group without sonification (control), a group observed for 1 day after sonification (C1), or a group observed for 7 days after sonification (C7). Of note, no clotting was induced. Each of these groups included three monkeys. Additionally, two middle-aged rhesus monkeys (18 years old) were sonicated after intravenous injection of alteplase (0.9 mg/kg) and followed for 7 days after procedure (R). Next, researchers applied therapeutic ultrasound beams to the right middle cerebral artery through a transducer overlying an acoustic temporal window (average temporal bone thickness of 1.72 mm for cynomolgus group). The therapeutic ultrasound beam generator used for sonothrombolysis (frequency 490 kHz, intensity 0.72 W/cm^2^) was alternated with a diagnostic color-flow imaging ultrasound beam for 15 min protocol blocks, repeated four times over the course of 60 min. Animals were euthanized after 24 h for the control and C1 groups, and after 7 days for the C7 and R groups. Their brains were histologically examined. The research team concluded that there was no evidence of histological tissue damage or neurological abnormalities caused by this system, nor evidence of an intracranial cavitation effect. Given the similarities between nonhuman primates and human brains, the lack of adverse acoustic effects in monkeys suggests that intracranial sonothrombolysis performed through a temporal window may be a safe option for treating acute ischemic stroke and other intracranial conditions in pediatric patients.

Recently, Kleven et al. investigated the effects of combining transcranial 120-kHz ultrasound with tPA therapy in a juvenile porcine model of intracerebral hemorrhage with concomitant thrombi, with direct infusion of autologous blood into the porcine white matter [21]. After excluding pigs with intraventricular or subdural hemorrhages, 32 pigs were included in the analysis in the following way: 9 did not receive intervention, 10 received tPA treatment only, and 13 received tPA and sonothrombolysis treatment. Investigators evaluated the effect of these therapies on thrombus density. They discovered that ultrasound had no enhancing effects on thrombolysis or hematoma evacuation, presumably because no exogenous cavitation nuclei were present due to the absence of microbubble administration. It has been described that employing an ultrasound contrast agent boosts cavitation activity and may be necessary to enhance thrombolysis [92,93,94].

Studies conducted in rat models have revealed that sonothrombolysis enhances recanalization rates in mesenteric microvessels and lowers cerebral infarct volumes compared with tPA therapy alone. When combined with sonothrombolysis, a dose reduction of tPA was appreciated [3,22].

Studies conducted in rabbit models using microbubbles combined with ultrasound showed promising results in treating ischemic stroke [20,59]. Culp et al. described that the use of different types of microbubbles with ultrasound resulted in significantly lower infarct volumes compared with controls. Similarly, Brown et al. found that combining microbubbles with rtPA yielded effective loss of clot with lower rtPA doses, and infarct volumes were reduced. In line with these findings, a porcine model demonstrated that combining an ultrasound contrast agent with low-frequency ultrasound improved the effectiveness of rtPA thrombolysis in accelerating clot resolution.

### 7.2. Extracranial Thrombosis

Two rabbit model studies examined the potential of combining microbubbles and ultrasound to improve thrombolytic therapies for inferior vena cava (IVC) thrombosis [65,95]. Gao et al. demonstrated that guided longer-pulse ultrasound, in conjunction with intraclot infusion of microbubbles, has the potential to achieve more prompt clot removal and lower the dose of a required thrombolytic agent, which would in turn, decrease the amount of thrombolysis-related side effects [95]. In the second in vivo rabbit IVC thrombosis model, Wang et al. examined a catheter-based thrombolytic device that employed low-frequency ultrasound and microbubbles loaded with fibrin-targeted drugs. The results demonstrated that 30 min of microbubble-enhanced sonothrombolysis with endovascular low-frequency ultrasound is equally effective as solely pharmacologic treatment. Wang et al. also found that, with the addition of fibrin-targeted drug-loaded bifunctional microbubbles, the fibrinolytic agent dosage was reduced by 60% [65]. Hence, these innovative approaches show promising potential for application in pediatric cases, providing a safer and efficient alternative for treating IVC thrombosis in children.

### 7.3. Vascular and Cardiovascular Conditions

The effects of microbubble-enhanced sonothrombolysis in boosting endothelial nitric oxide release for restoration of peripheral flow due to catheter-induced vasospasm (CIV) were studied by Kutty and colleagues in a porcine model [68]. A total of 24 randomized CIV treatments were performed in 16 pigs of unknown ages. Investigators found a significant reduction in vessel stenosis when ultrasound therapy was combined with microbubbles or alone compared with controls. They concluded that a brief application of diagnostic high MI impulses combined with commercially available microbubble infusion may be an effective noninvasive method for treating CIV. These findings are applicable to the pediatric population, as CIV, vascular injury, and thrombosis are common complications of femoral arterial catheterization in children. Children with congenital heart diseases often undergo multiple cardiac and vascular catheterizations and, at their baseline, hold a higher risk for CIV and thrombosis. Moreover, infants demonstrate an increased risk of thromboembolism, with up to 20% of thrombi occurring in the femoral vessels, likely in part due to small body size [68]. Despite advances in technology, arterial injury and thromboembolic events in critically ill catheterized children continue to be associated with high mortality and morbidity rates, with one autopsy study reporting umbilical venous catheter (UVC) thromboembolic events in 20 to 65% of neonates with a UVC in place at the time of demise [96,97,98,99].

In a pig model, a new therapy for acute coronary micro-thromboembolism was investigated [100]. Pig age was not reported. Researchers used targeted low-intensity ultrasound together with acoustic phase change nanoparticles. Since this treatment strategy resulted in reduced infarct sizes, improved left ventricular ejection fraction, and almost full recanalization of the culprit coronary branch, the authors indicated the possible clinical utility of this combination.

An intriguing study using a sheep model revealed a novel interventional sonothrombolysis technique for treating thrombosis in left ventricular assist devices [66]. This device injected drug-loaded microbubbles using ultrasound transducer rings mounted on the LVAD shell. Investigators discovered that this approach effectively removed the thrombus within 30 min, confirming its effectiveness and safety for potential therapeutic uses in the future. These findings could be applicable to children, as ventricular assist devices thrombosis has been described to occur in 18% of pediatric patients with a paracorporeal pulsatile device [101].

Two canine trials provided further information on the potential of ultrasound-based therapy for cardiovascular diseases. First, Mott et al. showed that ultrasound therapy could improve wall thickness and myocardial blood flow in ischemic tissue [84]. Similarly, Li et al. investigated the use of ultrasound and microbubbles in the treatment of microvascular thrombosis-induced coronary no-reflow and showed that the strategy efficiently dissolved microthrombi, improving myocardial blood flow, cardiac function, and infarct size [85].

## 8. Clinical Studies

Multiple studies examined sonothrombolysis in humans and in human models. Similar to animal models, most of these studies focus on application to the adult population, although application to pediatric populations should be considered. The most common conditions evaluated are myocardial infarction, ischemic stroke, pulmonary embolism, deep venous thrombosis, and peripheral arterial occlusion.

### 8.1. Myocardial Infarction

The vast majority of myocardial infarction sonothrombolysis studies focus on ST elevation myocardial infarction (STEMI), a cardiac event in which myocardial ischemia results in injury or necrosis of the myocardium. It is estimated that approximately 3 million people each year experience STEMI [102]. Although STEMI mortality has decreased dramatically over the past decade secondary to improvements in in-hospital care, STEMI morbidity remains a major concern. In a small 2021 pilot study, three STEMI patients underwent sonothrombolysis with microbubbles (Luminity, 1.5 mL diluted in 50 mL NaCl), using a Philips CX-50 ultrasound machine, while in ambulance transport to a percutaneous coronary intervention (PCI) center. In one case, sonothrombolysis, in addition to regular prehospital care, resulted in reperfusion before arrival at the PCI center. No arrhythmias or vital disturbances were observed directly after sonothrombolysis [103]. Another study randomized STEMI patients to a control PCI-only group or an experimental group consisting of sonothrombolysis with intravenous infusion of perflutren microbubbles (Definity, 3 mL as a 5% dilution) before and after emergent percutaneous coronary intervention [12]. The study found that ST-segment resolution occurred in 32% of the sonothrombolysis group and in only 4% of the PCI-only control group prior to PCI. Further, the sonothrombolysis group post-PCI demonstrated a reduced infarct size compared with the PCI-only group. An additional study using myocardial echocardiography with high mechanical index ultrasound impulses and intravenous infusion of Definity microbubbles (1–2 mL/min prior to PCI and continued post-PCI for a total of 30 min) found that sonothrombolysis prior to primary PCI resulted in pre-PCI thrombolysis in 7 out of 15 STEMI patients (47%) [104]. After reperfusion, 14/15 patients achieved ST-segment resolution greater than 50% at 30 min post-PCI. Although these small studies demonstrate promising results, clinical trials with a larger number of patients are warranted to assess the safety and efficacy of sonothrombolysis in STEMI patients.

No myocardial infarction sonothrombolysis studies involving children were retrieved during our search. A potential application could be acute myocardial infarction in children with Kawasaki disease, given patients with a disease course complicated by giant coronary artery aneurysm are at high risk for myocardial infarction B [105]. Although antiplatelet therapy is recommended for all patients with Kawasaki syndrome, giant aneurysm thrombosis still occurs, given altered flow mechanics and decreased aneurysmal wall sheer stress [106]. Further, myocardial infarction in children with coronary artery anomalies and congenital heart disease could serve as another potential application [107].

### 8.2. Stroke

With an annual mortality rate of 5.5 million, stroke is the leading cause of death worldwide in adults. Of those who survive a cerebrovascular accident, approximately 50% are chronically disabled [108]. Although advances in thrombectomy and thrombolytic agents have improved patient outcomes, the economic and social burden of ischemic stroke remains high. Multiple adult human studies have hypothesized that sonothrombolysis improves functional outcomes in stroke patients, yet with mixed results. One large, multicenter, double-blind, phase 3, randomized controlled trial enrolled 335 patients in an intervention group and 341 patients in a control group [24]. The intervention group underwent sonothrombolysis with an operator-independent device within 30 min of an alteplase (thrombolytic agent) bolus. The authors concluded that, although sonothrombolysis was feasible and safe, no clinical benefit was seen at 90 days. Similarly, the NORS-SASS trial (Norwegian sonothrombolysis in acute stroke study) randomized 183 patients to contrast-enhanced sonothrombolysis or sham control groups [27]. In the intervention group, SonoVue contrast (10 mL) was infused using an infusion pump for approximately 30 min at 0.3 mL/min, while 2 MHz ultrasound was applied for 60 min. Neurological improvement at 24 h and function outcome at 90 days demonstrated no significant differences between the sonothrombolysis and control groups. Conversely, a subgroup analysis of the CLOTBUST trial found that more patients achieved complete recanalization with sonothrombolysis as opposed to tPA alone. No microbubbles were administered [25]. Of the 126 enrolled adult patients, those who received sonothrombolysis also demonstrated higher functional independence at 90 days. Symptomatic intracranial hemorrhage was similar in both groups.

No clinical pediatric stroke sonothrombolysis articles were retrieved during our search. Vulnerable pediatric populations, such as children with sickle cell disease, thrombophilia, and other prothrombotic states, secondary to malignancy, infections, and medications, may experience stroke [109]. Among neonates, thrombolytic agents are almost always contraindicated, given the high risk of catastrophic intracranial hemorrhage. Therefore, the mainstay of neonatal treatment consists of supportive measures, such as controlling seizures, correcting dehydration and anemia, and treating concomitant infections [110]. Although infants typically do not receive anticoagulation in the United States to treat acute ischemic infarct, anticoagulation is often used in other countries. One single-center Canadian study examined 83 neonates with cerebral sinovenous thrombosis, 29 of whom received either warfarin, standard heparin, or low molecular weight heparin. A total of 14% of neonates with pre-existing hemorrhage experienced major hemorrhage versus 0% of children without pretreatment intracranial hemorrhage. Early follow-up imaging showed thrombus propagation in 28% of infants without anticoagulation versus 4% with anticoagulation. Of note, propagation was associated with new venous infarcts in 10% of neonates [111]. These findings suggest that anticoagulation in conjunction with sonothrombolysis may be safe in neonates and older children. However, further investigation is needed before clinical application.

### 8.3. Pulmonary Embolism

After myocardial infarction and stroke, pulmonary embolism is the third most common cause of cardiovascular death. The current standard treatment of care consists of direct-acting oral anticoagulants, catheter-directed thrombolysis, and inpatient and postoperative prophylaxis [112]. A 20-study meta-analysis examining catheter-directed thrombolysis for acute high- and intermediate-risk pulmonary embolism in 1168 adult patients concluded that ultrasound-assisted thrombolysis might be more effective than standard catheter-directed thrombolysis alone [26]. Specifically, in the high-risk pulmonary embolism cohort, clinical success for the catheter-directed thrombolysis alone group was 70.8%, while the ultrasound catheter-directed thrombolysis group was 83.1%. Another large meta-analysis consisting of 1028 human subjects determined that sonothrombolysis significantly reduced mean pulmonary artery pressure, right to left ventricle diameter ratio, and computed tomography (CT) obstruction scores, with a lower risk of major bleeding events and similar death rates, compared with pulmonary embolism patients undergoing systemic thrombolytic treatment [113].

A single pediatric case report was found during the literature search. The case report, titled “Successful recanalization of thrombotic occlusion in pulmonary artery stent using sonothrombolysis”, detailed a 13-month-old girl with left pulmonary artery stent thrombosis followed by embolism in the setting of postoperative heart surgery for hypoplastic left heart syndrome. After failed medical thrombolysis, physicians and family agreed to trial compassionate-use sonothrombolysis with Sonovue microbubbles (0.1 mL each injection, 6 mL in total) at 1.7 MHz. Following 50 min of sonothrombolysis, angiography revealed flow to the main pulmonary artery branch and to the periphery of the lung, noting residual thrombi within the distal vasculature. Following sonothrombolysis, the patient’s systemic oxygen improved from 64% to 78% and, clinically, providers deemed the patient hemodynamically stable. This case report illustrates that microbubbles may be safe and life-saving in these populations [1].

### 8.4. Deep Venous Thrombosis and Peripheral Artery Occlusion

Venous thrombosis, including deep vein thrombosis (DVT) and pulmonary embolism, is a common event, occurring at an annual incidence of about 1 per 1000 adults. Predisposing risk factors include age, immobility, trauma, pregnancy, hormone use, malignancy, obesity, and inherited and acquired disorders of anticoagulation [114]. Like venous thrombosis, peripheral artery disease is a common debilitating disease that affected approximately 202 million people worldwide in 2010 [115]. One meta-analysis reviewed sonothrombolysis in the treatment of DVT using EKOS, an endovascular system used for ultrasound-assisted thrombolysis that augments ultrasonic waves to thin and separate fibrin strands, further increasing lytic dispersion of the thrombus [116]. Among the 512 patients, sonothrombolysis using the EKOS system resulted in substantial clot lysis in 77–100% of cases. No procedure-related pulmonary emboli were reported, although one procedure-related death was documented [117]. Similarly, another DVT clinical study comparing catheter-directed thrombolysis to microbubble-enhanced ultrasound thrombolysis using a 1 MHz air-backed transducer found that the microbubble sonothrombolysis intervention group demonstrated shorter thrombolysis times and a lower streptokinase (thrombolytic agent) dosage [118]. With respect to acute peripheral arterial occlusions, a single arm phase II trial applied microbubbles (Luminity, 6 mL total, dilated with 0.9% to 40 mL total) with local ultrasound to patients within the first hour after administration of intra-arterial thrombolytic therapy. The median thrombolysis time was 47.5 h, with improvements in short term ABI and pain scores. Moreover, no serious adverse events resulted [119]. No pediatric venous or arterial occlusion clinical studies were identified in our search. As mentioned above in the preclinical animal studies section, thromboembolic disease poses a serious threat to children. In the critical care setting, central venous catheters (CVCs) and arterial catheters are prone to clot development. In fact, CVCs serve as the single largest risk factor for pediatric venous thromboembolism (VTE) in all pediatric populations [120]. CVCs lead to thromboembolism by vascular trauma during insertion and through turbulent flow related to catheter positioning in the vessel [121]. In children with end-stage renal disease, CVCs are the most commonly employed device for hemodialysis access. CVC thrombosis and occlusion underlie the most common cause of catheter failure, necessitating exchange. Often, CVC thrombosis distorts the vessel architecture and leads to stenosis, which precludes future hemodialysis [122]. To prevent these adverse outcomes, most clinicians employ prophylactic measures. Mechanical prophylaxis consists of compression stockings and intermittent pneumatic compression devices, while pharmacological agents comprise systemic anticoagulation, fibrinolytics, and ethanol locks [120]. Similar to adults, treatment of thromboembolism in the pediatric patient population consists of mechanical thrombectomy and anticoagulation [123]. However, these drugs have side effects, such as major bleeding and anaphylactic-type reactions, and may require subcutaneous injections and frequent monitoring, warranting the development of new devices with fewer adverse outcomes, such as sonothrombolysis.

## 9. Safety Parameters

In addition to other safety parameters, such as thermal effects, providers must prioritize the mechanical index, especially when using sonothrombolysis with microbubbles. The mechanical index (MI) is an indication of an ultrasound beam creating cavitation-related bioeffects or mechanical damage, noting that gas-filled organs, such as lungs and intestines, are the most sensitive to the effects of cavitation. Because MI is inversely proportional to the frequency of the beam, higher frequencies possess a lower mechanical index. To decrease the MI in a study, a provider can decrease the ultrasound beam output or distance the focal zone from the transducer. The MI must be below 1.9 in the United States [36].

The vast majority of studies concluded sonothrombolysis to be a safe procedure when performed by a trained professional. For example, in a systematic review of sonothrombolysis in the treatment of DVT, 20/512 (3.9%) patients experienced major bleeding and only one procedure-related patient death was documented [117]. However, some studies theorize that, in addition to accelerating clot lysis, microbubble activation leads to blood barrier disruption and bleeding in animal models. A case-control study examining the risk of hemorrhagic transformation after microbubble-enhanced sonothrombolysis in acute stroke demonstrated early recanalization but a high rate of hemorrhagic transformation following microbubble administration in 296 adult patients. Despite this high rate of hemorrhagic transformation, the risk of symptomatic intracranial hemorrhage did not increase [124].

Similar to the adult population, intracranial hemorrhage is devastating to the pediatric brain. Neonates diagnosed with intracranial hemorrhage often present with seizures, bulging fontanelles, increasing head circumference, and apnea. In one study examining full-term infants with intracranial hemorrhage with associated parenchymal involvement, the mortality rate was as high as 24.5% [125]. Surviving children face complications, such as hydrocephalus, long-term brain injury, and developmental delay. Although sonothrombolysis may be a less invasive and inexpensive approach to stroke treatment in a neonate, the risk of fatal complications must be considered, and ultrasound safety parameters must be standardized.

Ethical considerations play a pivotal role in the success of a medical intervention. In the pediatric population, children rely on parental or surrogate consent and rarely have a voice in medical decision making. A guardian must decide if the benefits of a treatment outweigh the risks, while a child must live with the consequences. It is of the utmost importance that the provider has a firm understanding of the family’s psychosocial needs and medical literacy before proceeding with a novel procedure. Unfortunately, although the current research suggests that sonothrombolysis is safe in adults, there is a dearth of clinical studies that pertain to the pediatric population.

In order to ensure the safety of this vulnerable population, more in vitro and in vivo animal studies need to be performed. In vitro studies should utilize phantoms that replicate a pediatric patient as opposed to an adult. If sonothrombolysis proves to be a safe intervention through further preclinical research, lower-risk therapies should be trialed first, such as deep venous thrombosis treatment, given bleeding into a limb is far less catastrophic than bleeding into the brain, heart, or lungs.

## 10. Conclusions

Sonothrombolysis is an adaptable and evolving advanced ultrasound technology that can potentially improve management and outcomes in patients with thrombotic disease. In vitro and animal studies have shown the efficacy and safety of sonothrombolysis for clot dissolution without harming the surrounding tissues or causing other major adverse events. When combined with the use of microbubbles, sonothrombolysis could offer enhanced results that warrant further study in human beings in conditions such as deep venous thrombosis, peripheral vascular disease, ischemic stroke, myocardial infarction, and pulmonary embolism.

Even though there is paucity of the literature regarding the potential applications of this technique in the pediatric population, it could be considered as a potential noninvasive therapeutic approach in the context of suspected thrombotic disease in children, as it has demonstrated success in adults. It is important to consider that the prevalence of thrombotic events in the pediatric population is lower compared with adults; however, sonothrombolysis can complement the clinical workup algorithm when necessary. Other than the difference in the prevalence of diseases, special considerations in children include different anatomy and physiology that could pose challenges in the application of sonothrombolysis; for these reasons, further research studies that test the safety and efficacy and optimize the use of sonothrombolysis in young population are warranted.

## Figures and Tables

**Table 1:** Key articles.

Study Type	Title Author PMID	Ultrasound Parameters	Type of Sonothrombolysis	Comments
Preclinical In Vitro				
	Ultrasound-assisted laser thrombolysis with endovascular laser and high-intensity focused ultrasound. Jo et al. [15] PMID: 33280145	Frequency: 0.5 MHz Laser fluence levels: 0 mJ/cm^2^, 2 mJ/cm^2^, and 4 mJ/cm^2^	Ultrasound only	Endovascular laser combined with US at low power and pressure levels can achieve effective thrombolysis.
	Sonothrombolysis: the contribution of stable and inertial cavitation to clot lysis. Petit et al. [16] PMID: 25601463	Frequency: 1 MHz Acoustic pressures: 200, 350, and 1300 kPA	Combinations of tPA + US + MB	The combination of tPA + US + MB demonstrated that inertial cavitation (1300 kPA) enhanced fibrin degradation compared with tPA alone.
	Dynamic behavior of microbubbles during long ultrasound tone-burst excitation: mechanistic insights into ultrasound/microbubble-mediated therapeutics using high-speed imaging and cavitation detection. Chen et al. [17] PMID: 26603628	Frequency: 1 MHz Focal length: 42.5 mm Various peak negative pressures: 0.25, 0.5, 1.0, and 1.5 MPa	US + MB	Ongoing cavitation activity during long tone bursts may lead to additional therapeutic benefits.
	Inertial cavitation ultrasound with microbubbles improves reperfusion efficacy when combined with tissue plasminogen activator in an in vitro model of microvascular obstruction. Goyal et al. [18] PMID: 28395964	Pulsed US: 1 MHz IC: 1.0 MPa, 1000 cycles, 0.33 Hz Stable cavitation: 0.23 MPa, 20% duty cycle, 0.33 Hz	US + tPA + MB	These findings suggest an IC regime can be used with tPA to achieve a high degree of fibrinolysis for both venous and arterial type microthrombi.
	Acoustic and elastic properties of a blood clot during microbubble-enhanced sonothrombolysis: hardening of the clot with inertial cavitation. Auboire et al. [19] PMID: 34683859	1 MHz sinusoidal ultrasound waves 0.8 Hz pulse repetition frequency	tPA alone (3 μg/mL) US + MB tPA + US + MB	The combination of tPA and sonothrombolysis with microbubbles augments sound speed and clot hardening, which in turn decreases the penetration of thrombolytic drugs and their efficacy.
Preclinical animal				
	Efficacy of sonothrombolysis using microbubbles produced by a catheter-based microfluidic device in a rat model of ischemic stroke. Dixon et al. [3] PMID: 30689066	Frequency: 1 MHz Ultrasound energy was applied at a 10% duty factor in bursts of 100 cycles with a peak negative pressure of 500 kPa.	Group A: Control; Group B: 0.09 mg/kg tPA; Group C: 0.9 mg/kg tPA; Group D: 0.09 mg/kg tPA + US + MB.	Sonothrombolysis + US reduced cerebral infarct volumes by approximately 50% compared with no therapy, in addition to significantly improving functional neurological outcomes at 24 h and permitting tPA dose reduction by 3.3-fold compared with tPA alone.
	Successful microbubble sonothrombolysis without tissue-type plasminogen activator in a rabbit model of acute ischemic stroke. Culp et al. [20] PMID: 21700942	Frequency: 1 MHz Intensity: 0.8 W/cm^2^	Embolized without treatment (control) tPA only tPA + US perflutren lipid MB (0.16 mg/kg) + US albumin 3 μm MB (5 × 10⁹ MB) + US Tagged albumin 3 μm MB + US	Infarct volume percent was lower for rabbits treated with lipid MB + US, 3 μm MB + US, and tagged 3 μm MB + US compared with controls. Microbubble groups had lower infarct volumes than controls.
	Effect of recombinant tissue plasminogen activator and 120 kHz ultrasound on porcine intracranial thrombus density. Kleven et al. [21] PMID: 36336551	Frequency: 120 kHZ	tPA alone Ultrasound with tPA	No enhancement of tPA-mediated thrombolysis was noted with the addition of sonothrombolysis.
	Microbubble-mediated sonothrombolysis improves outcome after thrombotic micro-embolism-induced acute ischemic stroke. Lu et al. [22] PMID: 27048701	Frequency: 14 MHz MI = 0.17	tPA only group (10 mg/kg) Microbubble only (0.008 mL/min) Microbubble-enhanced (0.008 mL/min) + half-dose tPA group (5 mg/kg)	Microbubble-mediated sonothrombolysis significantly reduced cerebral infarction and improved neurological deficit (US + MB group).
	Ultrasound safety with midfrequency transcranial sonothrombolysis: preliminary study on normal Macaca monkey brain. Shimizu et al. [23] PMID: 22475695	Frequency = 490 kHz Intensity = 0.72 W/cm^2^	Two elder rhesus monkeys received 0.9 mg/kg of alteplase.	None of the monkeys showed neurologic deficits after sonothrombolysis.
Clinical studies				
	Sonothrombolysis in ST-segment elevation myocardial infarction treated with primary percutaneous coronary intervention. Mathias, Jr. et al. [12] PMID: 30894317	Control group: low MI (<0.2) Therapeutic group: 1.8 MHz 1.2 to 1.3 MI <5-μs pulse	Microbubble-enhanced	In the high MI sonothrombolysis group, there was sustained improvement in systolic function and reduced need for defibrillators at 6-month follow up.
	Safety and efficacy of sonothrombolysis for acute ischemic stroke: a multicenter, double-blind, phase 3, randomized controlled trial. Alexandrov et al. [24] PMID: 30878103	A 2 MHz pulsed-wave transcranial ultrasound for 120 min (total average power 32 mW; maximum spatial peak temporal average intensity 207 mW/cm^2^; pulse repetition frequency 8·3 kHz; pulse duration 5 μs)	Ultrasound in conjunction with intravenous alteplase (0.9 mg/kg)	No clinical benefit after 90 days was found.
	Outcomes following sonothrombolysis in severe acute ischemic stroke: subgroup analysis of the CLOTBUST trial. Barlinn et al. [25] PMID: 25079049	Frequency: 2 MHz	Ultrasound in conjunction with intravenous alteplase (0.9 mg/kg)	Researchers observed an absolute increase in 3-month functional independence rate with sonothrombolysis compared with the IV tPA only group.
	A meta-analysis of outcomes of catheter-directed thrombolysis for high- and intermediate-risk pulmonary embolism. Avgerinos et al. [26] PMID ID: 29909859	Variable	Ultrasound in conjunction with tPA (variable dosages)	Sonothrombolysis may be more effective than standard catheter-directed thrombolysis in the higher risk population.
	Successful recanalization of thrombotic occlusion in pulmonary artery stent using sonothrombolysis. Mathias, Jr. et al. [1] PMID: 30828677	Frequency: 1.7 MHz MI: 1.3 Pulse duration: 20 μs	Microbubble-enhanced (6 mL in total) Bolus of 0.05 mg/kg of alteplase followed by 24 h infusion at 5 mg/kg/hr (day prior to sonothrombolysis)	Sonothrombolysis with microbubbles resulted in recanalization of pulmonary artery obstruction in a child with congenital heart disease.
	NOR-SASS (Norwegian sonothrombolysis in acute stroke study): randomized controlled contrast-enhanced sonothrombolysis in an unselected acute ischemic stroke population. Nacu et al. [27] PMID: 27980128	Frequency: 2 MHz MI: <1.0.	Microbubble-enhanced (Sonovue, 10 mL) Tenecteplase 0.4 mg/kg or alteplase 0.9 mg/kg	No significant clinical effect of sonothrombolysis was demonstrated. The trial stopped prematurely, secondary to lack of funding.

MB = microbubbles; MI = mechanical index; US = ultrasound; tPA = tissue plasminogen activator; IC = inertial cavitation.

**Table 2:** Conditions evaluated in human studies.

Condition	Zone	Number of Patients Evaluated
Pulmonary Embolism	Asia	42
	Europe	217
	Middle East	441
	North America	1538
	South America ^a^	1
	Total	2239
Acute Ischemic Stroke	Asia	84
	Central America	1
	Europe	1184
	Middle East	42
	Multicentric ^b^	733
	North America	55
	South America	61
	Total	2160
Myocardial Infarction	Europe	23
	Middle East	141
	North America	196
	South America	400
	Total	760
Brain Hemorrhage	Europe	300
	North America	33
	Total	333
Deep Venous Thrombosis	Asia	50
	Europe	23
	Total	73
Peripheral Artery Occlusion	Asia	1
	Europe	44
	Total	45
Venous Graft Thrombosis	Middle East	20
	North America	1
	Total	21
Occluded Transjugularportosystemic Shunt	Europe	1
	Total	1
Grand Total	5632	

^a^ Study conducted in pediatric population. ^b^ Included centers located in ≥2 different countries.

## Data Availability

The data presented in this study are available on request from the corresponding author. The data are not publicly available due to privacy and ethical restrictions.
